# Chronic Kidney Disease in Primary Care: Outcomes after Five Years in a Prospective Cohort Study

**DOI:** 10.1371/journal.pmed.1002128

**Published:** 2016-09-20

**Authors:** Adam Shardlow, Natasha J. McIntyre, Richard J. Fluck, Christopher W. McIntyre, Maarten W. Taal

**Affiliations:** 1 Renal Unit, Royal Derby Hospital, Derby, United Kingdom; 2 Centre for Kidney Research and Innovation, Division of Medical Sciences and Graduate Entry Medicine, School of Medicine, The University of Nottingham, Royal Derby Hospital, Derby, United Kingdom; 3 Division of Nephrology, Schulich School of Medicine and Dentistry, University of Western Ontario, London, Ontario, Canada; Istituto Mario Negri, ITALY

## Abstract

**Background:**

Chronic kidney disease (CKD) is commonly managed in primary care, but most guidelines have a secondary care perspective emphasizing the risk of end-stage kidney disease (ESKD) and need for renal replacement therapy. In this prospective cohort study, we sought to study in detail the natural history of CKD in primary care to better inform the appropriate emphasis for future guidance.

**Methods and Findings:**

In this study, 1,741 people with CKD stage 3 were individually recruited from 32 primary care practices in Derbyshire, United Kingdom. Study visits were undertaken at baseline, year 1, and year 5. Binomial logistic regression and Cox proportional hazards models were used to model progression, CKD remission, and all-cause mortality. We used Kidney Disease: Improving Global Outcomes (KDIGO) criteria to define CKD progression and defined CKD remission as the absence of diagnostic criteria (estimated glomerular filtration rate [eGFR] >60 ml/min/1.73 m^2^ and urine albumin-to-creatinine ratio [uACR] <3 mg/mmol) at any study visit. Participants were predominantly elderly (mean ± standard deviation (SD) age 72.9 ± 9.0 y), with relatively mild reduction in GFR (mean ± SD eGFR 53.5 ± 11.8 mL/min/1,73 m^2^) and a low prevalence of albuminuria (16.9%). After 5 y, 247 participants (14.2%) had died, most of cardiovascular causes. Only 4 (0.2%) developed ESKD, but 308 (17.7%) evidenced CKD progression by KDIGO criteria. Stable CKD was observed in 593 participants (34.1%), and 336 (19.3%) met the criteria for remission. Remission at baseline and year 1 was associated with a high likelihood of remission at year 5 (odds ratio [OR] = 23.6, 95% CI 16.5–33.9 relative to participants with no remission at baseline and year 1 study visits). Multivariable analyses confirmed eGFR and albuminuria as key risk factors for predicting adverse as well as positive outcomes. Limitations of this study include reliance on GFR estimated using the Modification of Diet in Renal Disease study (MDRD) equation for recruitment (but not subsequent analysis) and a study population that was predominantly elderly and white, implying that the results may not be directly applicable to younger populations of more diverse ethnicity.

**Conclusions:**

Management of CKD in primary care should focus principally on identifying the minority of people at high risk of adverse outcomes, to allow intervention to slow CKD progression and reduce cardiovascular events. Efforts should also be made to identify and reassure the majority who are at low risk of progression to ESKD. Consideration should be given to adopting an age-calibrated definition of CKD to avoid labelling a large group of people with age-related decline in GFR and low associated risk as having CKD.

## Abstract 2

In this prospective cohort study, Adam Shardlow and colleagues report on the progression of chronic kidney disease over 5 years among patients in primary care.

## Author Summary

**Why Was This Study Done?:**

- Chronic kidney disease affects 10%–20% of adults in most countries and is associated with multiple adverse outcomes, including increased risk of death, progression to end-stage kidney disease (requiring dialysis or kidney transplantation), and increased risk of diseases of the heart and arteries.
- The risk of these adverse outcomes varies considerably, and previous studies indicate that most people with chronic kidney disease are at low risk.
- Many previous studies have been conducted in large teaching hospitals and have understandably emphasized the risk of end-stage kidney disease, but the results may not be applicable to the majority with chronic kidney disease because most have mild disease, are cared for by family doctors, and are never referred to a kidney specialist.
- We conducted this study to better understand the risks associated with chronic kidney disease in people cared for by family doctors in order to provide a perspective that is applicable to the majority of people affected.

**What Did the Researchers Do and Find?:**

- We individually assessed 1,741 people with mild (stage 3) chronic kidney disease at 32 family doctors’ clinics and reassessed them after 1 and 5 y.
- We found that most people (34%) had stable kidney function, only a very small minority (4 people or 0.2%) developed end-stage kidney disease, and 18% evidenced less severe progression after 5 y.
- Surprisingly, kidney function improved in some people (19%) to the extent that they no longer had evidence of chronic kidney disease.

**What Do These Findings Mean?:**

- Our data emphasize that the management of chronic kidney disease by family doctors should focus on identifying the minority of people who are at high risk of adverse outcomes for more intensive treatment and referral to a kidney specialist.
- People at low risk should also be identified so that they can be reassured and spared unnecessary treatment or referral.
- An internationally agreed definition for “remission” of chronic kidney disease is needed so that this outcome can be studied in more detail in other populations.
- Our findings may not be directly applicable to populations with younger age or greater ethnic diversity.

## Introduction

In the UK and many other countries, the majority of people with chronic kidney disease (CKD) are diagnosed and managed in primary care clinics without ever being referred to a nephrologist. In contrast, most detailed studies investigating the risks associated with CKD have been led by nephrologists, resulting in a predominantly secondary care perspective. Consequently, guidelines based on these studies tend to emphasize the risk of end-stage kidney disease (ESKD) and the need for timely referral to facilitate preparation for renal replacement therapy. Landmark epidemiological studies have highlighted the importance of diagnosing CKD because the abnormalities that define CKD (reduced glomerular filtration rate [GFR] and albuminuria) are powerful independent risk factors for multiple adverse outcomes, including progression of CKD, development of ESKD [1,2], acute kidney injury (AKI) [3], excess cardiovascular events (CVEs) [4], and increased mortality [5]. Nevertheless, the prognosis associated with CKD is extremely heterogeneous, and the risk of adverse outcomes varies widely according to the population studied. For example, population-based studies have reported that the majority of people with CKD are at low risk of developing ESKD [6], whereas studies of people known to have CKD and managed in secondary care report ESKD as a common outcome [7,8].

Many previous studies have understandably focused on risks associated with CKD [1–5]. However, it is arguably as important to study positive outcomes such as stable CKD or remission of CKD so that people at low risk for adverse outcomes can be spared unnecessary intervention and referral to nephrology clinics. Interestingly, there is currently no consensus on when CKD should be considered no longer present (in remission), and there is therefore a lack of readily comparable data to indicate how frequently remission occurs or the factors that contribute to it.

The majority of people with CKD in primary care are elderly, and albuminuria is present in only a minority [9], suggesting that overall this population is at low risk for progression to ESKD. In this prospective cohort study, we sought to study in detail the natural history of CKD in primary care to better inform the appropriate emphasis for future guidance on caring for people with CKD in a primary care setting.

## Methods

### Ethics

The Renal Risk in Derby (RRID) study was approved by the Nottingham Research Ethics Committee 1. All participants provided written, informed consent. The RRID study complies with the Declaration of Helsinki and the principles of Good Clinical Practice.

### Participants

**Fig 1:**
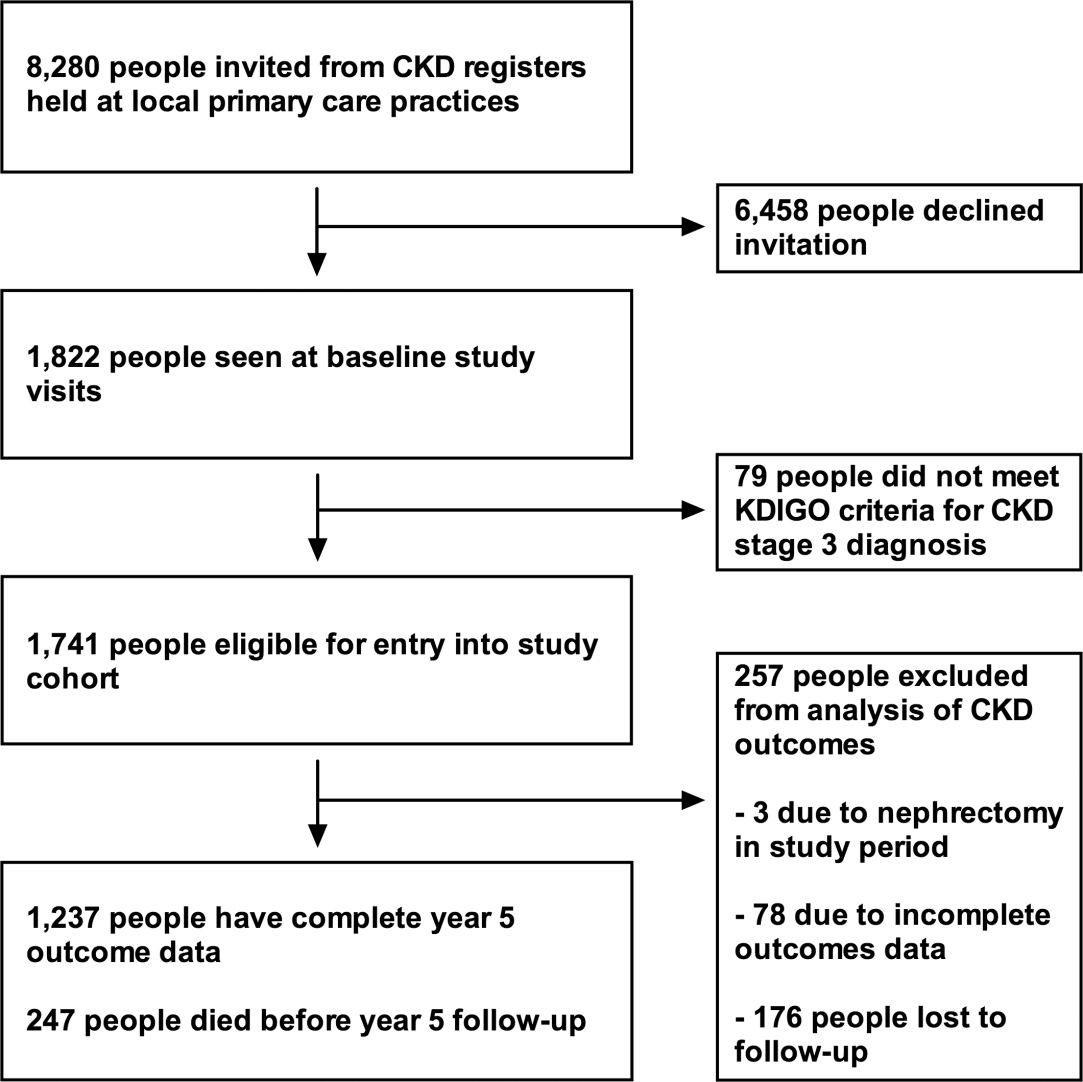
Flowchart showing study participant outcomes and follow-up.

Detailed methods for the RRID study have been published previously [9]. In brief, participants were individually recruited from 32 primary care clinics in Derbyshire, UK, between 2008 and 2010 and prospectively studied. In total, 8,280 people were invited from registers of people with CKD stage 3, 1,822 attended baseline visits, and 1,741 were eligible to participate (Fig 1). Participants were aged >18 y and at least two estimated GFR (eGFR) results (derived from the Modification of Diet in Renal Disease study [MDRD] equation) of 30–59 ml/min/1.73 m^2^, more than 90 d apart, were required to be eligible [10]. People judged to have a life expectancy of less than 1 y, unable to attend study visits, or with a solid organ transplant were excluded.

### Study Visits

Visits were conducted at baseline and repeated at 1 and 5 y. Prior to each visit, participants completed a background questionnaire covering demographic variables and social, medical, and medication history. Questionnaires were reviewed at study visits and clarified as required. Height, weight, and waist and hip circumference were measured. Three blood pressure measurements that differed by <10% were taken using an oscillometric device (UA-767 Plus 30, A&D Medical) after at least 5 min rest.

### Laboratory Methods

Participants were asked to abstain from eating meat for 12 h prior to study visits to avoid confounding the serum creatinine assay. Blood and urine samples were analysed in a single clinical laboratory at the Royal Derby Hospital for standard haematological and biochemical variables. Creatinine was measured using the Jaffe method, standardized against an isotope dilution mass spectrometry method. GFR was estimated using the Chronic Kidney Disease Epidemiology Collaboration (CKD-EPI) equation in the primary analysis, with an additional sensitivity performed using the MDRD equation. The average urine albumin-to-creatinine ratio (uACR) from three consecutive early morning specimens was used for analysis.

Thirty-one participants who were unable to attend for year 5 study visits were able to submit blood and urine samples specifically for the study. For 176 participants, unable to either attend for a study visit or to submit study samples, we incorporated blood and uACR results taken for clinical purposes into the dataset. These were selected to be less than 6 mo from the year 5 visit due date. Samples taken during hospital admissions were not used.

### Mortality Data

Date and cause of death as stated on death certificates were obtained from the Office of National Statistics via the Health and Social Care Information Centre (HSCIC). Independent of each other and without knowledge of other participant data, three investigators (AS, RJF, and MWT) assessed the information on death certificates and classified cause of death into four categories (cardiovascular, malignancy, infection, and other). Differences in classification were subsequently resolved by discussion.

### Endpoint Definitions

We used Kidney Disease: Improving Global Outcomes (KDIGO) definitions for CKD, CKD staging, and CKD progression [10]. Progression of CKD was defined as a 25% decline in GFR, coupled with a worsening of GFR category, or an increase in albuminuria category. Our prespecified endpoint from study design (development of ESKD or doubling of serum creatinine) was not used because of a very small number of events noted during year 5 follow-up. The KDIGO definition was used as an internationally agreed standard of CKD progression that was observed more frequently in our study population. We defined CKD remission as the presence of both eGFR >60 ml/min/1.73 m^2^ and uACR <3 mg/mmol at any study visit in an individual who had previously met KDIGO diagnostic criteria for CKD.

### Statistical Analysis

Analysis was performed using IBM SPSS version 22. Urine ACR was logarithmically transformed prior to multivariable analysis. Outcomes were analysed using binomial logistic regression models. For analyses of progression and remission, participants with a complete year 5 outcome (eGFR and uACR values or a date of death before the year 5 visit) were included. Basic models were initially produced including baseline age, eGFR log uACR, and sex. Variables that were significantly associated with the outcome on univariable analysis were then added in groups, according to biological and clinical relevance. The final models included all variables that were significantly associated with the outcome in previous models and gave the best discrimination. For each model, predicted probabilities were used to draw receiver operating characteristic (ROC) curves, and area under the ROC curve (AUROC) was used to compare models. Survival analysis was performed using Cox proportional hazards models. All 1,741 participants were included in the survival analysis. Death prior to a year 5 visit was considered an event. Participants were censored at date of year 5 visit, date of first dialysis, or 5 y from the time of baseline visit if neither of these events occurred. Three participants underwent unilateral nephrectomy during the study follow-up period and therefore were excluded from analysis of CKD progression and remission.

## Results

### Cohort Description and Outcomes

Description of the cohort at baseline has previously been published [9]. Key baseline variables are given in Table 1 for the whole cohort and groups according to outcome. Data on renal outcomes at 5 y were obtained in 1,484 participants (85.2%), and survival data were available in all participants (from the Office of National Statistics). Two hundred and forty-seven of 1,741 people (14.2%) died prior to their year 5 visit, representing an age-standardised mortality rate per year of 4.2% in males and 2.2% in females. This compares to a UK age-standardised mortality rate of 3.9% and 2.9% in males and females, respectively [11]. Four participants (0.2%) reached ESKD, and 308 (17.7%) evidenced progression of CKD by KDIGO criteria. However, 593 participants (34.1%) had stable CKD, and 336 (19.3%) met our criteria for CKD remission (Table 1). Year 5 outcomes are reported in Table 2 and summarised in S1 Fig. Outcomes are further subdivided by baseline CKD category in Table 3 and S2 Fig.

**Table 1:** Basic cohort descriptive statistics and breakdown by year 5 outcome.

Variable (*n*)	Total (1,741)	Stable CKD (593)	CKD Remission (336)	CKD Progression (308)	Died (247)	Lost to Follow-up (257)
Female Sex (%)	1,052 (60.4)	350 (59.0)	252 (75.0)	160 (51.9)	104 (42.1)	186 (72.4)
Age (years)	72.9 ± 9.0	72.6 ± 8.1	67.1 ± 8.5	73.3 ± 8.7	78.7 ± 6.9	74.9 ± 9.3
eGFR–CKD-EPI (ml/min/1.73 m^2^)	53.5 ± 11.8	51.6 ± 9.6	64.1 ± 9.0	50.3 ± 11.8	46.3 ± 10.0	55.1 ± 12.0
eGFR–MDRD (ml/min/1.73 m^2^)	52.5 ± 10.4	50.7 + 8.7	60.9 ± 7.8	49.7 ± 10.9	47.1 ± 95	54.2 ± 10.4
uACR (mg/mmol)	0.3 (0.0–1.5)	0.3 (0.0–1.4)	0.1 (0.0–0.4)	0.9 (0.2–2.1)	1.1 (0.1–3.9)	0.3 (0.0–1.3)
Diabetes (%)	294 (16.9)	93 (15.7)	30 (8.9)	78 (25.3)	57 (23.1)	36 (14.0)
CVD (%)	387 (22.2)	107 (18.0)	49 (14.6)	70 (22.7)	99 (40.1)	62 (24.1)
Current or Previous Smoker (%)	947 (54.4)	307 (51.8)	159(47.3)	173 (56.2)	165(66.8)	143 (55.6)
ACE/ARB use (%)	1,123 (64.5)	374 (63.1)	186 (55.4)	222 (72.1)	173 (70.0)	168 (65.4)
Weight (kg)	78.2 ± 15.5	79.5 ± 14.3	77.6 ± 15.5	79.2 ± 15.8	775 ± 175	75.8 ± 15.6
BMI (kg/m^2^)	29.0 ± 5.1	29.5 ± 4.9	29.0 ± 5.2	28.9 ± 4.9	28.3 ± 5.4	29.0 ± 5.4
Waist:Hip Ratio	0.91 ± 0.09	0.91 ± 0.08	0.88 ± 0.09	0.92 ± 0.09	0.94 ± 0.09	0.90 ± 0.09
SBP (mmHg)	134.0 ± 18.3	133.7 ± 17.1	130.0 ± 16.8	136.9 ± 17.4	135.7 ± 22.0	134.8 ± 195
DBP (mmHg)	72.8 ± 11.0	73.2 ± 11.0	74.7 ± 10.7	72.2 ± 10.6	70.2 ± 11.6	72.8 ± 11.0
Haemoglobin (g/dl)	13.2 ± 1.4	13.3 ± 1.3	13.6 ± 1.2	12.9 ± 15	12.8 ± 15	13.2 ± 15
Corrected Calcium (mmol/l)	2.38 ± 0.10	2.38 ± 0.10	2.38 ± 0.09	2.37 ± 0.10	2.36 ± 0.10	2.39 ± 0.12
Phosphate (mmol/l)	1.11 ± 0.18	1.10 ± 0.18	1.11 ± 0.18	1.12 ± 0.17	1.11 ± 0.18	1.11 ± 0.18
Albumin (g/l)	40.7 ± 3.2	40.8 ± 2.9	41.4 ± 2.9	40.4 ± 3.6	39.6 ± 35	40.6 ± 3.0
Bicarbonate (mmol/l)	25.5 ± 2.7	25.6 ± 25	26.0 ± 2.5	24.9 ± 2.7	25.4 ± 3.1	255 ± 2.7
Total Cholesterol (mmol/l)	4.8 ± 1.19	4.8 ± 1.2	5.0 ± 1.2	4.7 ± 1.3	4.4 ± 1.1	4.8 ± 1.1
Urate (μmol/l)	384 ± 91	391 ± 89	334 ± 79	402 ± 85	409 ± 100	374 ± 92.8
**Change in Given Variables at Different Time Points**						
Change in CKD-EPI eGFR at Y1	−0.74 ± 7.8	−0.35 ± 7.4	0.40 ± 8.4	−2.7 ± 7.7	−0.81 ± 8.1	−0.73 ± 7.6
Change in SBP at Y1 (mmHg)	−3.2 ± 16.0	−3.4 ± 14.6	−1.8 ± 8.6	−4.1 ± 17.1	−5.0 ± 18.8	−2.1 ± 18.3
Change in DBP at Y1 (mmHg)	−2.4 ± 9.3	−2.6 ± 9.1	−1.7 ± 8.6	−2.7 ± 9.3	−2.8 ± 10.0	−2.2 ± 105
Change in uACR at Y1	0.17 (−0.1 to 0.6)	0.15 (−0.1 to 0.4)	0.17 (0.0–0.4)	0.4 (−0.0 to 1.6)	0.3 (−1.1 to 1.3)	0.2 (−0.1 to 0.7)
Change in Weight at Y5 (kg)	−1.6 ± 5.9	−1.7 ± 6.2	−1.1 ± 5.4	−1.9 ± 5.9	N/A	N/A

Normally distributed variables are presented as mean ± standard deviation (SD). Non-normally distributed variables are presented as median (interquartile range). Categorical variables are presented as a number (percentage). ACE, angiotensin-converting enzyme; ARB, angiotensin receptor blocker; BMI, body mass index; CVD, cardiovascular disease; DBP, diastolic blood pressure; N/A, not applicable; SBP, systolic blood pressure; Y1, year 1; Y5, year 5.

**Table 2:** Year 5 outcomes and associated independent predictors.

	Year 5 Outcome			
	Stable CKD	CKD Remission	CKD Progression	Died before Year 5
**Number of Participants (%)**	593 (34.1%)	336 (19.3%)	308 (17.7%)	247 (14.2%)
**Independent Predictors**		Higher eGFR	Lower eGFR	Lower eGFR
		Lower Age		Greater Age
		Lower uACR	Higher uACR	Higher uACR
			Male Gender	Male Gender
			Lower Haemoglobin	Lower Haemoglobin
			Lower Bicarbonate	Higher Bicarbonate
				Lower Albumin
				Previous CVD
			Diabetes	
		Greater Increase in eGFR over 1 y	Greater Loss of eGFR over 1 y	

**Table 3:** Numbers of participants reaching each year 5 outcome by baseline KDIGO CKD GFR and albuminuria category.

		**CKD Remission**				**Stable CKD**	
	**A1**	**A2**	**A3**		**A1**	**A2**	**A3**
**G1/G2**	236 (47.5%)	2 (6.3%)	1 (16.7%)	**G1/G2**	102 (20.8%)	14 (43.8%)	3 (50%)
**G3a**	85 (13.0%)	5 (4.3%)	0 (0%)	**G3a**	268 (40.9)	51 (43.6%)	5 (33.3%)
**G3b**	6 (2.2%)	1 (1.1%)	0 (0%)	**G3b**	96 (35.3%)	39 (43.8%)	8 (34.8%)
**G4**	0 (0%)	0 (0%)	0 (0%)	**G4**	4 (21.1%)	4 (28.6%)	0 (0%)
		**CKD Progression**				**All-Cause Mortality**	
	**A1**	**A2**	**A3**		**A1**	**A2**	**A3**
**G1/G2**	60 (12.1%)	3 (9.4%)	1 (16.7%)	**G1/G2**	18 (3.6%)	6 (18.8%)	0 (0%)
**G3a**	114 (17.4%)	25 (21.4%)	2 (13.3%)	**G3a**	88 (13.4%)	23 (19.7%)	4 (26.7%)
**G3b**	69 (25.7%)	15 (16.9%)	9 (39.1%)	**G3b**	59 (21.7%)	30 (33.7%)	5 (21.7%)
**G4**	8 (42.1%)	2 (14.3%)	0 (0%)	**G4**	7 (36.8%)	5 (35.7%)	2 (100%)

G1 to G4 refer to KDIGO GFR categories in the classification of CKD stage. G1 ≥90 ml/min/1.73 m^2^, G2 60–90 ml/min/1.73 m^2^, G3a 45–60 ml/min/1.73 m^2^, G3b 30–45 ml/min/1.73 m^2^, G4 15–30 ml/min/1.73 m^2^. A1 to A3 refer to KDIGO albuminuria (uACR) categories in the classification of CKD stage. A1 ≤3 mg/mmol, A2 3–30 mg/mmol, A3 >30 mg/mmol.

### Associations with Progression of CKD

A basic multivariable model that included baseline eGFR, sex, uACR, and age as predictors of CKD progression was associated with an AUROC of 0.69 (95% CI 0.66–0.74). The addition of baseline haemoglobin, bicarbonate, diabetes status, and systolic blood pressure (SBP) produced a model with an AUROC of 0.73 (95% CI 0.69–0.76). In this model, baseline age was not statistically significant. The impact of the change at 1 y in SBP, diastolic blood pressure (DBP), and eGFR was also assessed. Change in eGFR at year 1 was a significant determinant of CKD progression, but change in SBP and DBP at 1 y did not enter the model (Table 4). When the recently validated four-variable Kidney Failure Risk Equation (KFRE) [2,12] was applied to our study population at baseline, the majority were assessed to be at extremely low risk (median 5-y probability of ESKD = 0.08%). In the four participants who did progress to ESKD, predicted 5-y risks at baseline were 54.0%, 33.1%, 14.9%, and 9.0%, respectively.

**Table 4:** Univariable and multivariable associations of CKD progression at 5 y.

Variable	Univariable Odds Ratio (95% CI)	Multivariable Odds Ratio (95% CI)					
		Model 1 (Basic Model)	Model 2	Model 3	Model 4	Model 5	Model 6 (Best Model)
eGFR	0.59 (0.51–0.68)*	0.72 (0.62–0.84)*	0.75 (0.64–0.87)*	0.85 (0.71–1.02)	0.74 (0.63–0.86)*	0.82 (0.70–0.97)*	0.71 (0.60–0.85)*
Age	1.41 (1.22–1.62)*	1.25 (1.07–1.45)*	1.25 (1.07–1.45)*	1.22 (1.04–1.44)*	1.12 (0.95–1.32)	1.17 (0.98–1.38)	1.12 (0.94–1.34)
Male Sex	1.70 (1.31–2.21)*	1.24 (0.94–1.64)	1.23 (0.92–1.64)	1.61 (1.15–2.25)*	1.25 (0.94–1.66)	1.50 (1.10–2.03)*	1.48 (1.08–2.02)*
Log uACR	1.76 (1.53–2.04)*	1.59 (1.37–1.85)*	1.56 (1.34–1.81)*	1.59 (1.36–1.86)*	1.56 (1.35–1.82)*	1.53 (1.31–1.79)*	1.50 (1.28–1.75)*
Haemoglobin	0.68 (0.60–0.79)*			0.71 (0.60–0.85)*		0.73 (0.62–0.86)*	0.75 (0.64–0.89)*
Phosphate	1.11 (0.97–1.26)			1.04 (0.89–1.13)			
Corrected Calcium	0.78 (0.76–1.00)			0.97 (0.83–0.96)			
Bicarbonate	0.71 (0.62–0.82)*			0.83 (0.71–0.96)*		0.83 (0.72–0.96)*	0.83 (0.71–0.96)*
Albumin	0.83 (0.73–0.95)*			0.98 (0.84–1.14)			
Total Cholesterol	0.89 (0.78–1.02)			1.10 (0.95–1.27)			
Urate (μmol/l)	1.37 (1.20–1.56)*			1.09 (0.92–1.29)			
Diabetes	2.22 (1.62–3.06)*		1.71 (1.22–2.40)*			1.53 (1.07–2.19)*	1.52 (1.06–2.20)*
Previous CVD	1.46 (1.06–2.00)*		1.14 (0.82–1.60)				
Current or Previous smoker	1.27 (0.98–1.65)		1.05 (0.79–1.39)				
SBP	1.32 (1.15–1.51)*				1.30 (1.09–1.55)*	1.22 (1.02–1.46)*	1.17 (0.98–1.41)
DBP	0.86 (0.76–0.99)*				0.98 (0.68–0.96)*	0.96 (0.80–1.15)	0.97 (0.80–1.16)
BMI	0.92 (0.80–1.05)						
Waist:Hip Ratio	1.33 (1.16–1.51)*						
Y1 Change eGFR	0.70 (0.61–0.81)*						0.63 (0.54–0.75)*
Y1 Change SBP	0.92 (0.80–1.05)						
Y1 Change DBP	0.96 (0.84–1.10)						
AUROC (95% CI)		0.69 (0.66–0.73)	0.70 (0.67–0.73)	0.72 (0.69–0.75)	0.70 (0.67–0.73)	0.73 (0.69–0.76)	0.74 (0.71–0.78)

AUROC, area under receiver operator characteristic curve. The eGFR was calculated using the CKD-EPI formula, and all variables were measured at baseline unless stated. Odds ratios are expressed per 1 SD increase in the independent variable.

* *p*-value < 0.05

### Associations with All-Cause Mortality

Cause of death was classified as cardiovascular in 94 cases (38.1% of 247 deaths), malignancy in 63 (25.5%), infection in 50 (20.2%), “other” in 34 (13.8%), and “no data available” in 6 (2.4%). Two people died before year 5 follow-up but after the development of ESKD. Participants who died tended to be older than other outcome groups and had lower eGFR and higher uACR on average at baseline (Table 1). Cox proportional hazards models identified age, male sex, baseline eGFR, log uACR, haemoglobin, albumin, and bicarbonate as independent predictors of death (Table 5). Change in eGFR at year 1 did not enter the model (hazard ratio [HR] = 1.00, *p* = 0.67).

**Table 5:** Cox proportional hazards models: Hazard ratios for all-cause mortality at 5 y.

Variable	Univariable Relative Hazard (95% CI)	Multivariable Relative Hazard (95% CI)					
		Model 1	Model 2	Model 3	Model 4	Model 5	Model 6
eGFR	0.50 (0.44–0.57)*	0.69 (0.60–0.80)*	0.70 (0.60–0.81)*	0.73 (0.61–0.87)*	0.71 (0.61–0.82)*	0.68 (0.57–0.82)*	0.62 (0.62–0.84)*
Age	2.47 (2.10–2.89)*	2.01 (1.70–2.38)*	1.96 (1.66–2.22)*	1.93 (1.62–2.30)*	1.99 (1.68–2.37)*	2.14 (1.76–2.61)*	1.90 (1.60–2.25)*
Male Sex	2.20 (1.71–2.84)*	1.62 (1.25–2.10)*	1.46 (1.12–1.91)*	1.87 (1.38–2.54)*	1.67 (1.29–2.17)*	1.81 (1.34–2.45)*	1.89 (1.44–2.49)*
Log uACR	1.57 (1.37–1.81)*	1.30 (1.12–1.50)*	1.24 (1.07–1.44)*	1.22 (1.05–1.41)*	1.31 (1.13–1.52)*	1.26 (1.07–1.49)*	1.22 (1.06–1.41)*
Haemoglobin	0.70 (0.62–0.80)*			0.83 (0.72–0.97)*			0.84 (0.74–0.96)*
Phosphate	1.00 (0.88–1.13)			1.00 (0.87–1.16)			
Corrected Calcium	0.86 (0.75–0.98)*			0.97 (0.85–1.11)			
Bicarbonate	0.97 (0.85–1.10)			1.16 (1.02–1.32)*			1.17 (1.03–1.32)*
Albumin	0.73 (0.65–0.81)*			0.79 (0.70–0.91)*			0.82 (0.72–0.93)*
Total Cholesterol	0.68 (0.59–0.78)*			0.90 (0.77–1.05)			
Urate	1.33 (1.18–1.50)*			1.02 (0.88–1.17)			
Diabetes	1.56 (1.16–2.09)*		1.25 (0.92–1.68)				1.20 (0.88–1.63)
Previous CVD	2.62 (2.03–3.38)*		1.84 (1.42–2.38)*				1.81 (1.39–2.35)*
Current or Previous Smoker	1.76 (1.35–2.29)*		1.27 (0.97–1.68)				
SBP	1.10 (0.97–1.25)				0.97 (0.84–1.12)		
DBP	0.76 (0.67–0.86)*				0.90 (0.77–1.05)		
BMI	0.84 (0.74–0.96)*						
Waist:Hip Ratio	1.39 (1.23–1.57)*						
Y1 Change eGFR	1.00 (0.86–1.16)					0.94 (0.79–1.11)	
Y1 Change SBP	0.89 (0.77–1.04)						
Y1 Change DBP	0.96 (0.83–1.12)						

The eGFR was calculated using the CKD-EPI equation. All variables were measured at baseline unless stated. Relative hazards are expressed per 1 SD increase in the independent variable.

* *p*-value < 0.05

### Associations with CKD Remission

At baseline, participants who evidenced remission at year 5 had a higher mean eGFR and a lower median uACR, compared to the rest of the study cohort (Table 1). In addition, participants with remission were younger and evidenced a higher proportion of females (Table 6). A basic multivariable model, including age, sex, eGFR, and log uACR as independent variables, was associated with an AUROC of 0.85 (95% CI 0.82–0.87) (Table 6). This was improved to 0.86 (95% CI 0.84–0.88) by addition of the change in eGFR seen at 1 y and baseline haemoglobin. There was no significant difference in weight change between renal outcome groups (Table 1), nor did weight change enter multivariable models as a predictor of remission.

**Table 6:** Univariable and multivariable associations of CKD remission at 5 y.

Variable	Univariable Odds Ratio (95% CI)	Multivariable Odds Ratio (95% CI)					
		Model 1 (Basic Model)	Model 2	Model 3	Model 4	Model 5	Model 6 (Best Model)
eGFR	4.92 (3.99–6.06)*	4.07 (3.27–5.07)*	4.01 (3.21–5.00)*	3.80 (2.99–4.83)*	4.18 (3.34–5.24)*	5.25 (4.09–6.73)*	5.10 (3.97–6.55)*
Age	0.50 (0.44–0.58)*	0.67 (0.56–0.79)*	0.66 (0.56–0.79)*	0.70 (0.59–0.84)*	0.69 (0.57–0.83)*	0.75 (0.63–0.90)*	0.76 (0.64–0.91)*
Female Sex	2.30 (1.74–3.04)*	1.21 (0.87–1.69)	1.22 (0.87–1.71)	1.44 (0.96–2.18)	1.16 (0.83–1.63)	1.16 (0.83–1.63)	1.34 (0.92–1.94)
Log uACR	0.60 (0.52–0.68)*	0.67 (0.57–0.79)*	0.67 (0.57–0.79)*	0.68 (0.57–0.80)*	0.67 (0.57–0.79)*	0.69 (0.58–0.81)*	0.69 (0.58–0.81)*
Haemoglobin	1.33 (1.17–1.53)*			1.22 (1.00–1.49)*			1.20 (0.99–1.45)
Phosphate	1.01 (0.89–1.15)			1.04 (0.88–1.23)			
Corrected Calcium	1.08 (0.95–1.23)			0.98 (0.83–1.15)			
Bicarbonate	1.32 (1.16–1.51)*			1.17 (0.98–1.38)			
Albumin	1.30 (1.14–1.49)*			1.16 (0.97–1.38)			
Total Cholesterol	1.23 (1.09–1.39)*			0.94 (0.80–1.11)			
Urate	0.52 (0.45–0.61)*			0.96 (0.79–1.17)			
Diabetes	0.42 (0.28–0.63)*		0.79 (0.49–1.28)				
Previous CVD	0.70 (0.50–0.99)*		1.12 (0.73–1.70)				
Current or Previous smoker	0.79 (0.61–1.01)		0.97 (0.71–1.32)				
SBP	0.74 (0.64–0.85)*				0.87 (0.70–1.08)		
DBP	1.18 (1.04–1.35)*				0.95 (0.78–1.16)		
BMI	0.95 (0.83–1.08)						
Waist:Hip Ratio	0.68 (0.60–0.78)*						
Y1 Change eGFR	1.22 (1.07–1.38)*					1.71 (1.45–2.01)*	1.70 (1.44–2.00)*
Y1 Change SBP	1.13 (0.99–1.30)						
Y1 Change DBP	1.11 (0.97–1.26)						
AUROC (95% CI)		0.85 (0.82–0.87)	0.85 (0.82–0.87)	0.85 (0.82–0.87)	0.85 (0.82–0.87)	0.86 (0.84–0.88)	0.86 (0.84–0.88)

The eGFR was calculating using the CKD-EPI equation, and all variables were measured at baseline unless stated. Odds ratios are expressed per 1 SD increase in the independent variable.

* *p*-value < 0.05

**Fig 2:**
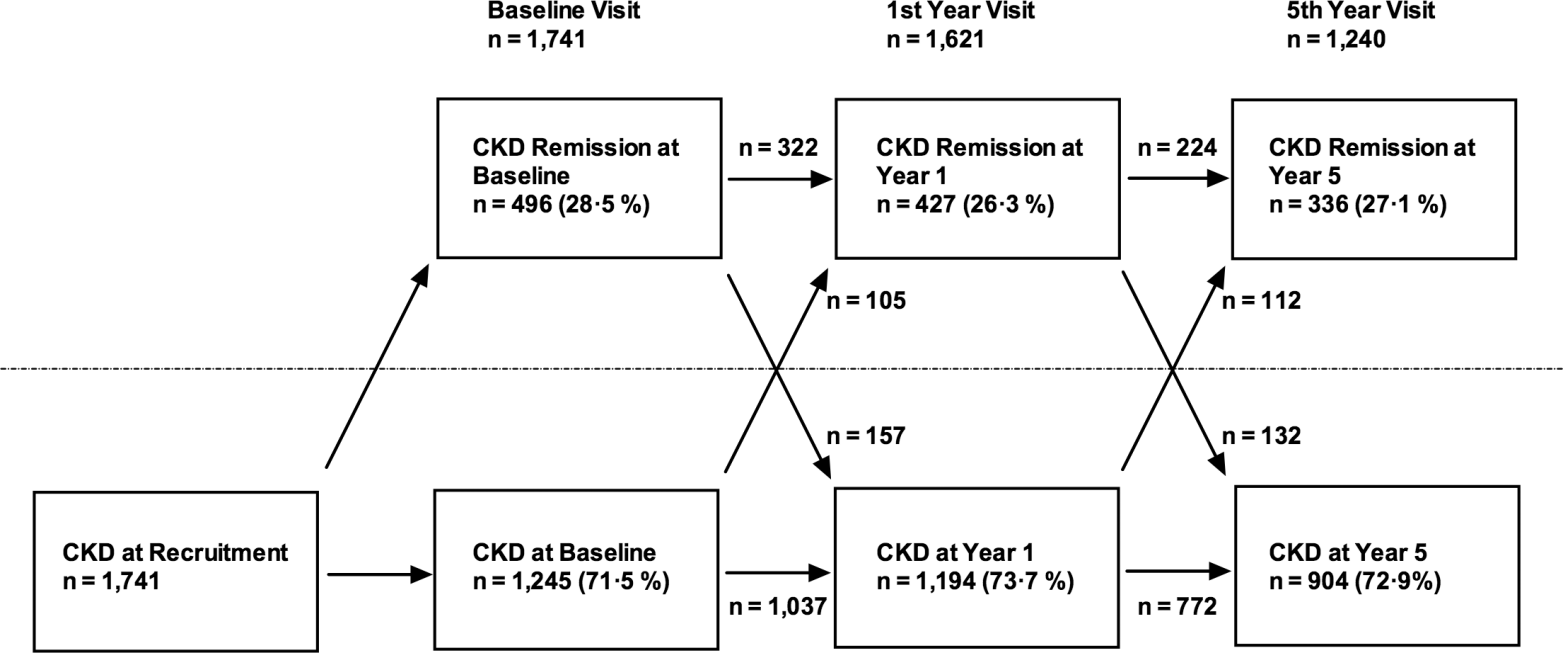
Flowchart showing numbers of participants demonstrating CKD and CKD remission at each study visit. Arrows indicate numbers of participants transitioning from one condition to the other.

The number of participants at each time point who demonstrated CKD remission is illustrated in Fig 2. Despite meeting KDIGO diagnostic criteria for CKD stage 3 prior to study entry, 496 participants (28.5%) no longer met the criteria for a diagnosis of CKD at the baseline study visit. Of this group, 224 (45.2%) remained in remission at year 5. Remission at 5 y was most likely in the group with remission at both baseline and year 1 (odds ratio relative to those with no remission at baseline and year 1 = 23.6, 95% CI 16.5–33.9, *p* < 0.001). Those with remission at baseline only (OR = 5.9, 95%, CI 3.8–9.2, *p* < 0.001) and year 1 only (OR = 7.1, 95% CI 4.3–11.8, *p* < 0.001) showed intermediate likelihood of remission at 5 y. The group with remission at baseline and year 1 also evidenced lower mortality (3.2%) over 5 y compared with those with remission at baseline only (5.0%) and those with remission at year 1 only (5.3%). Mortality was significantly lower in all groups demonstrating remission at any time point compared to the group who met criteria for CKD at both baseline and year 1 (15.7%; log-rank test *p* < 0.001).

### Sensitivity Analysis

Repeat analyses using the MDRD formula to estimate GFR revealed similar numbers of participants reaching each outcome. Predictors of different outcomes were similar using MDRD eGFR to those presented above using CKD-EPI eGFR (S1–S4 Tables).

## Discussion

In this population with CKD stage 3 in primary care, the majority of whom did not meet criteria for referral to a nephrologist [13], we observed a very low incidence of ESKD over 5 y (0.2%), and only a minority evidenced progression of CKD as defined by KDIGO (17.7%). Similar to population-based studies, the risk of all-cause (14.2%) and cardiovascular mortality (5.4%) greatly exceeded the risk of ESKD [1,14]. Stable CKD (34.1%) was more common than progression, and a substantial minority evidenced remission of CKD (19.3%).

Our data confirm in a primary care population the previous findings that reduced GFR and albuminuria are important independent risk factors for adverse outcomes [1–4]. Other risk factors were similar to those reported in previous studies [15]. In univariable analysis, diabetes was a significant predictor of CKD progression and mortality. After multivariable adjustment, diabetes remained an independent predictor only of CKD progression. Nevertheless, as diabetes was a predictor of CKD progression, it may have adversely affected survival indirectly through its effect on GFR. Lower haemoglobin independently predicted both CKD progression and all-cause mortality [16]. As reported recently by the Chronic Renal Insufficiency Cohort (CRIC) study investigators, lower serum bicarbonate was associated with CKD progression [17]. Serum albumin was an independent predictor of mortality, but not CKD progression. Change in GFR at 1 y was an independent determinant of CKD progression at 5 y. This observation confirms that annual assessment of GFR is useful for monitoring and as a marker of prognosis even in people with relatively mild CKD. Overall, the risk of progression to ESKD was extremely low over 5 y, and less severe CKD progression was observed in only a minority. The KFRE successfully identified four participants who developed ESKD as moderate or high risk, but risk prediction tools that predict risk over longer periods or give an estimate of “time to ESKD” may be more useful in primary care.

Despite globally applied criteria for the diagnosis of CKD, there is no consensus on when CKD should be considered no longer present (in remission). Therefore, there is a lack of readily comparable data to indicate how frequently remission occurs or the factors that contribute to it. Using a definition based on lack of any diagnostic criteria for CKD, we observed remission in a substantial proportion of people who met the KDIGO criteria for CKD prior to the baseline study visit. We propose that this observation was in part due to diagnostic considerations. We recruited participants based on previous routine GFR values estimated using the MDRD equation. Prior to study visits, participants were asked not to eat meat for 12 h, and for analysis, the more accurate CKD-EPI formula (published in 2009, after initiation of the study) was used to estimate GFR. Thus, our data illustrate the importance of applying the most accurate equation to estimate GFR and avoidance of meat prior to testing (previous studies have reported that meat ingestion can increase the serum creatinine by as much as 20 μmol/L) [18]. Nevertheless, there were participants who met the diagnostic criteria for CKD at the baseline visit who evidenced remission at 1 and 5 y (Fig 2). We propose that changes in medication or hydration status and healing of mild kidney damage may have contributed to this remission. A further possibility is that loss of muscle mass with increasing age may result in a decrease in serum creatinine and a rise in eGFR, but this does not seem to have been a contributory factor since weight change was not associated with remission in this cohort. Improvement in eGFR over time has been reported previously in 41% of participants in a large database study (median 7 ml/min/1.73 m^2^ improvement over 2 y) [19]. This cohort had a similar mean age (76.1 y) to our study, but these participants did not necessarily meet formal definitions for CKD and included some people with an eGFR >60 ml/min/1.73 m^2^.

Together, these data make a strong case for an internationally agreed definition of CKD remission so that it can be studied in other populations. Furthermore, a definition of remission will allow some patients to be removed from CKD registers, which may have implications for their health and life insurance. We propose that remission should be defined (in persons previously diagnosed with CKD) as the absence of diagnostic criteria for CKD that persists for at least 1 y, since this was associated with a substantially higher likelihood of long-term remission than a single set of normal values. Remission at any time point was also associated with lower mortality risk. We have identified lower age, higher eGFR, and lower uACR as predictors of remission with good discrimination, and these simple variables may therefore be used to identify patients who should be reassessed for remission or who should perhaps be given a provisional rather than a firm diagnosis of CKD. It remains to be shown, however, whether those meeting CKD criteria for a short period prior to improvement carry any residual excess risk.

The benign prognosis observed in participants in CKD category G3a A1 raises the question of whether older people with mildly reduced GFR but no albuminuria should be labelled as having CKD at all. This is important because our data confirm that G3a A1 represents the largest group of those diagnosed with CKD in primary care (S2 Fig). It has been suggested that an age-calibrated definition of CKD should be applied to avoid labelling people with age-related GFR decline and low risk of adverse outcomes as having CKD. Based on an analysis of values in normal populations across the age spectrum and the risks associated with reduced GFR, it has been proposed that the GFR threshold for diagnosis of CKD in the absence of albuminuria in those over 65 y should be lowered from 60 to 45 ml/min/1.73 m^2^ [20]. Our data support this recommendation, though it should be noted that progression of CKD over 5 y was observed in 17.4% of those with G3a A1 at baseline, suggesting that monitoring of GFR and uACR in this group should continue.

### Strengths and Limitations

Notable strengths of this study include use of strict KDIGO criteria for diagnosis of CKD stage 3 prior to study entry and detailed clinical characterisation of participants at each study visit. In this analysis, we have focussed on basic risk factors that would be easily measurable in clinical settings, but the use of novel biomarkers may improve risk prediction in the future.

Our study included predominantly white people, reflecting the demographic composition of the population of Derbyshire. In addition, the study population was elderly (mean age 72.9 ± 9.0 y), reflecting the higher prevalence of CKD in older people. Although our study population was generally representative of people with CKD cared for in primary care in the UK [21], the lack of ethnic diversity and relatively high age may limit application of our findings to populations with different ethnic composition or younger age. Recruitment commenced in 2008, and we therefore used the MDRD equation to diagnose CKD. The MDRD equation is known to underestimate GFR at higher levels of ≥60 ml/min/1.73 m^2^ and is less accurate than the CKD-EPI equation [22]. It is possible, therefore, that some participants were included who would not have been diagnosed with CKD were the CKD-EPI equation to have been used. Nevertheless, our study reflects a “real-world” situation with some people still having a diagnosis of CKD based on the MDRD equation. Sensitivity analyses using GFR estimated using the MDRD equation produced similar results to the primary analysis.

In this analysis, we used KDIGO definitions for CKD progression. The study protocol specified a definition of ESKD or doubling of serum creatinine as markers of progression, but these endpoints were met too infrequently to be useful for analysis of our cohort. Nevertheless, we believe that analysis using less severe CKD progression as an endpoint remains relevant because it has been shown that those with greater declines in eGFR are at higher future risk of ESKD and all-cause mortality [1].

### Conclusions

We have observed, in a primary care setting, that the most common outcome associated with CKD stage 3 over 5 y was stable kidney function. Moreover, a significant minority of people evidenced CKD remission. Our data therefore suggest that management of CKD in primary care should focus principally on identifying the minority of people who are at high risk of adverse outcomes for intervention to slow CKD progression and reduce CVEs. Efforts should also be made to identify and reassure the majority who are at low risk of progression to ESKD. Consideration should be given to adopting an age-calibrated definition of CKD to avoid labelling a large group of people with age-related decline in GFR and low associated risk as having CKD. Nevertheless, robust mechanisms should be in place to identify the minority at high risk for developing ESKD to facilitate timely referral.

## Supporting Information

S1 STROBE Checklist Study Strengthening the Reporting of Observational Studies in Epidemiology (STROBE) checklist. (DOCX)

S1 Fig Breakdown of study cohort by outcome. Independent predictors for CKD remission, CKD progression, and mortality before year 5 are given in boxes. (TIFF)

S2 Fig Breakdown of outcomes by baseline KDIGO CKD stage. (TIFF)

S1 Protocol Current study protocol. (DOC)

S1 Table Outcomes after 5 y, using the MDRD equation to calculate eGFR. (DOC)

S2 Table Univariable and multivariable associations with CKD progression, using the MDRD equation to calculate eGFR. (DOCX)

S3 Table Univariable and multivariable associations with 5-y all-cause mortality, using the MDRD equation to calculate eGFR. (DOCX)

S4 Table Univariable and multivariable associations of CKD remission, using the MDRD equation to calculate eGFR. (DOCX)

## Abbreviations

AKI: acute kidney injury
AUROC: area under the ROC curve
BMI: body mass index
CKD: chronic kidney disease
CKD-EPI: Chronic Kidney Disease Epidemiology Collaboration
CRIC: Chronic Renal Insufficiency Cohort
CVD: cardiovascular disease
CVE: cardiovascular event
DBP: diastolic blood pressure
eGFR: estimated glomerular filtration rate
ESKD: end-stage kidney disease
HR: hazard ratio
HSCIC: Health and Social Care Information Centre
KDIGO: Kidney Disease: Improving Global Outcomes
KFRE: Kidney Failure Risk Equation
MDRD: Modification of Diet in Renal Disease study
OR: odds ratio
ROC: receiver operating characteristic
RRID: Renal Risk in Derby
SBP: systolic blood pressure
SD: standard deviation
STROBE: Strengthening the Reporting of Observational Studies in Epidemiology
uACR: urine albumin-to-creatinine ratio
