# Supplementary figures and images for: Chronic Kidney Disease in Primary Care: Outcomes after Five Years in a Prospective Cohort Study
Source: PLoS Med. 2016 Sep 20;13(9):e1002128. doi: 10.1371/journal.pmed.1002128 (PMC5029805; doi:10.1371/journal.pmed.1002128)

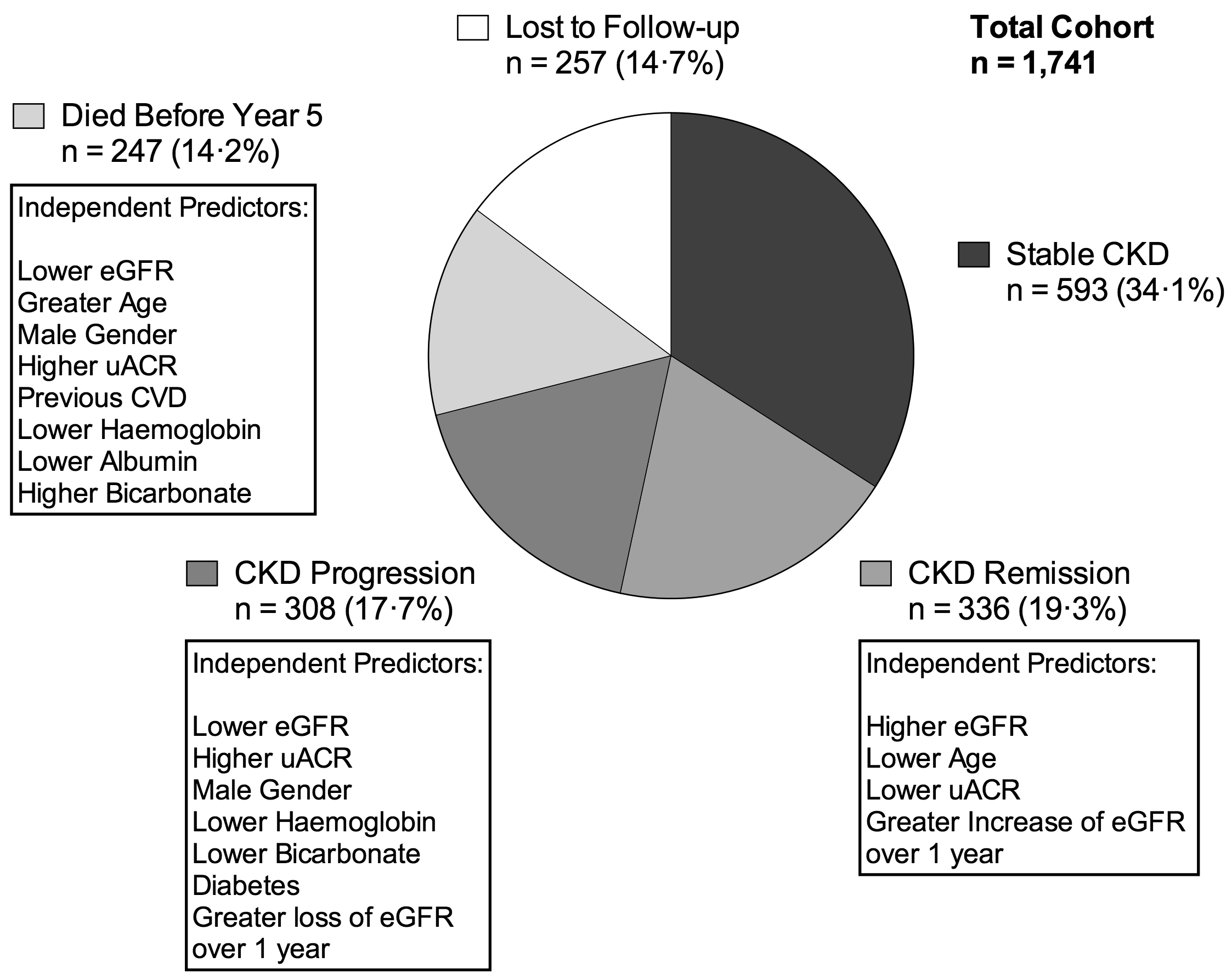

Supplement: S1 Fig — (TIFF) [file pmed.1002128.s002.tiff]

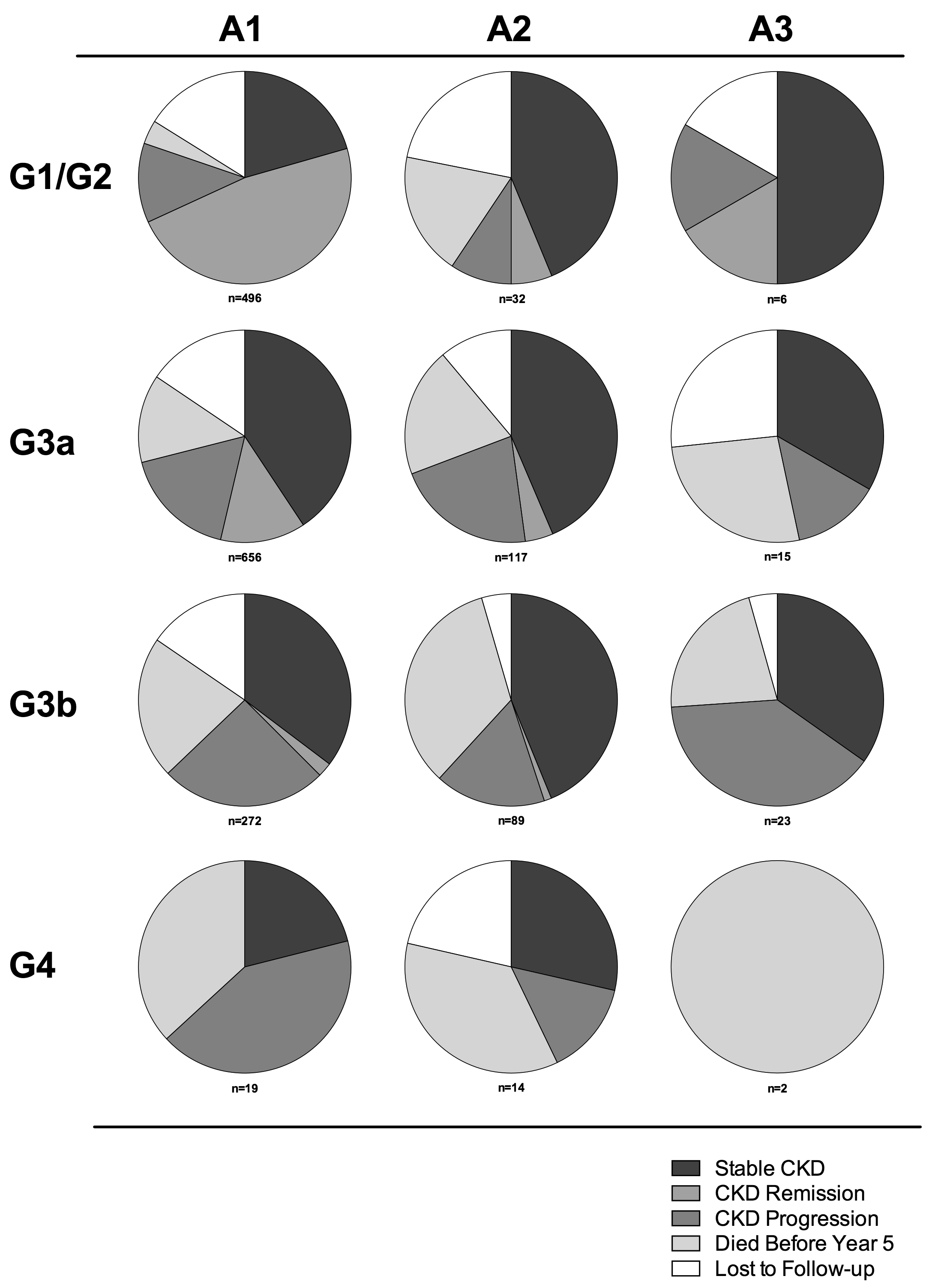

Supplement: S2 Fig — (TIFF) [file pmed.1002128.s003.tiff]
