# Supplementary material for: Chronic Kidney Disease in Primary Care: Outcomes after Five Years in a Prospective Cohort Study
Source: PLoS Med. 2016 Sep 20;13(9):e1002128. doi: 10.1371/journal.pmed.1002128 (PMC5029805; doi:10.1371/journal.pmed.1002128)
Supplement: S1 Table — (DOC) [file pmed.1002128.s005.doc]

S1 Table : Outcomes after 5 years, using the MDRD equation to calculate eGFR

| **Outcome** | **Stable CKD** | **CKD Remission** | **CKD Progression** | **Died before Year 5** | **Lost to Follow up** |
| --- | --- | --- | --- | --- | --- |
| **Number (%)** | 649  (37.3) | 297  (17.1) | 291  (16.7) | 247  (14.2) | 257  (14.8) |
