# Supplementary material for: Chronic Kidney Disease in Primary Care: Outcomes after Five Years in a Prospective Cohort Study
Source: PLoS Med. 2016 Sep 20;13(9):e1002128. doi: 10.1371/journal.pmed.1002128 (PMC5029805; doi:10.1371/journal.pmed.1002128)
Supplement: S2 Table — (DOCX) [file pmed.1002128.s006.docx]

| Variable | Univariable  Odds Ratio  (95% CI) | Multivariable Odds Ratio  (95% CI) | | | | | |
| --- | --- | --- | --- | --- | --- | --- | --- |
|  |  | Model 1  (Basic Model) | Model 2 | Model 3 | Model 4 | Model 5 | Model 6  (Best Model) |
| eGFR | 0.73 (0.64–0.83)* | 0.82 (0.71–0.94)* | 0.83 (0.72–0.95)* | 0.94 (0.80–1.11) | 0.82 (0.72–0.95)* | 0.90 (0.78–1.04) | 0.79 (0.68–0.92)* |
| Age | 1.10 (0.97–1.26) | 1.02 (0.89–1.17) | 1.03 (0.90–1.18) | 1.00 (0.87–1.16) | 0.94 (0.81–1.09) | 0.97 (0.83–1.12) | 0.97 (0.84–1.14) |
| Male Gender | 1.32 (1.02–1.70)* | 1.08 (0.82–1.41) | 1.11 (0.84–1.47) | 1.20 (0.87–1.65) | 1.09 (0.83–1.43) | 1.16 (0.87–1.55) | 1.18 (0.88–1.58) |
| Log uACR | 1.54 (1.34–1.77)* | 1.43 (1.24–1.66)* | 1.41 (1.22–1.63)* | 1.43 (1.23–1.67)* | 1.40 (1.21–1.63)* | 1.36 (1.17–1.59)* | 1.35 (1.16–1.58)* |
| Haemoglobin | 0.77 (0.68–0.88)* |  |  | 0.84 (0.71–0.98)* |  | 0.84 (0.72–0.98)* | 0.85 (0.73–1.00)* |
| Phosphate | 1.10 (0.97–1.25) |  |  | 1.05 (0.91–1.22) |  |  |  |
| Corrected Calcium | 0.90 (0.78–1.03) |  |  | 0.94 (0.81–1.09) |  |  |  |
| Bicarbonate | 0.72 (0.63–0.82)* |  |  | 0.80 (0.69–0.92)* |  | 0.80 (0.70–0.92)* | 0.81 (0.71–0.94)* |
| Albumin | 0.89 (0.79–1.01) |  |  | 1.00 (0.87–1.15) |  |  |  |
| Total Cholesterol | 0.95 (0.84–1.08) |  |  | 1.07 (0.93–1.24) |  |  |  |
| Urate | 1.24 (1.09–1.41)* |  |  | 1.10 (0.95 -1.29) |  |  |  |
| Diabetes | 1.87 (1.38–2.54)* |  | 1.56 (1.13–2.15)* |  |  | 1.36 (0.97–1.90) | 1.40 (0.99–1.97) |
| Previous CVD | 0.96 (0.70–1.31) |  | 0.80 (0.57–1.10) |  |  |  |  |
| Current or ex -smoker | 1.06 (0.82–1.37) |  | 0.94 (0.72–1.24) |  |  |  |  |
| SBP | 1.21 (1.06–1.37)* |  |  |  | 1.24 (1.06–1.45)* | 1.19 (1.01–1.40)* | 1.16 (0.99–1.37) |
| DBP | 0.93 (0.82–1.06) |  |  |  | 0.85 (0.73–1.00)* | 0.95 (0.80–1.13) | 0.95 (0.80–1.13) |
| BMI | 0.97 (0.85–1.10) |  |  |  |  |  |  |
| Waist:Hip Ratio | 1.20 (1.06–1.36)* |  |  |  |  |  |  |
| Y1 Change eGFR | 0.72 (0.63–0.83)* |  |  |  |  |  | 0.69 (0.59–0.81)* |
| Y1 Change SBP | 0.97 (0.85–1.11) |  |  |  |  |  |  |
| Y1 Change DBP | 0.97 (0.85–1.11) |  |  |  |  |  |  |
| p value <0.05*  eGFR calculated using MDRD formula, all variables measured at baseline unless stated  Odds ratios are expressed per 1 standard deviation increase in the independent variable | | | | | | | |

S2 Table : Univariable and Multivariable associations with CKD progression using the MDRD equation to calculate eGFR
