# Supplementary material for: Chronic Kidney Disease in Primary Care: Outcomes after Five Years in a Prospective Cohort Study
Source: PLoS Med. 2016 Sep 20;13(9):e1002128. doi: 10.1371/journal.pmed.1002128 (PMC5029805; doi:10.1371/journal.pmed.1002128)
Supplement: S3 Table — (DOCX) [file pmed.1002128.s007.docx]

S3 Table : Univariable and Multivariable associations with 5 year all cause mortality using the MDRD equation to calculate eGFR

| Variable | Univariable  Relative Hazard (95% CI) | Multivariable Relative Hazard  (95% CI) | | | | | |
| --- | --- | --- | --- | --- | --- | --- | --- |
|  |  | Model 1 | Model 2 | Model 3 | Model 4 | Model 5 | Model 6 |
| eGFR | 0.57 (0.50–0.64)* | 0.70 (0.60–0.81)* | 0.73 (0.63–0.83)* | 0.76 (0.65–0.89)* | 0.74 (0.64–0.84)* | 0.72 (0.61–0.84)* | 0.75 (0.65–0.86)* |
| Age | 2.47 (2.10–2.89)* | 1.96 (1.66–2.22)* | 2.07 (1.76–2.44)* | 2.03 (1.71–2.40)* | 2.10 (1.78–2.48)* | 2.27 (1.88–2.75)* | 1.99 (1.69–2.36)* |
| Male Gender | 2.20 (1.71–2.84)* | 1.46 (1.12–2.19)* | 1.53 (1.17–1.99)* | 1.94 (1.43–2.64)* | 1.75 (1.35–2.26)* | 1.90 (1.41–2.57)* | 1.97 (1.50–2.59)* |
| Log uACR | 1.57 (1.37–1.81)* | 1.30 (1.13–1.50)* | 1.24 (1.08–1.44)* | 1.22 (1.05–1.41)* | 1.31 (1.14–1.52)* | 1.26 (1.07–1.49)* | 1.22 (1.06–1.41)* |
| Haemoglobin | 0.70 (0.62–0.80)* |  |  | 0.83 (0.72–0.96)* |  |  | 0.84 (0.74–0.96)* |
| Phosphate | 1.00 (0.88–1.13) |  |  | 1.00 (0.87–1.16) |  |  |  |
| Corrected Calcium | 0.86 (0.75–0.98)* |  |  | 0.97 (0.85–1.11) |  |  |  |
| Bicarbonate | 0.97 (0.85–1.10) |  |  | 1.16 (1.02–1.32)* |  |  | 1.17 (1.03–1.32)* |
| Albumin | 0.73 (0.65 -0.81)* |  |  | 0.79 (0.70–0.91)* |  |  | 0.82 (0.72–0.93)* |
| Total Cholesterol | 0.68 (0.59–0.78)* |  |  | 0.90 (0.77–1.05) |  |  |  |
| Urate | 1.33 (1.18–1.50)* |  |  | 1.02 (0.88–1.18) |  |  |  |
| Diabetes | 1.56 (1.16–2.09)* |  | 1.25 (0.92–1.69) |  |  |  | 1.20 (0.88–1.64) |
| Previous CVD | 2.62 (2.03–3.38)* |  | 1.84 (1.42–2.38)* |  |  |  | 1.81 (1.39–2.35)* |
| Ever smoker | 1.76 (1.35–2.29)* |  | 1.27 (0.97–1.68) |  |  |  |  |
| SBP | 1.10 (0.97–1.25) |  |  |  | 0.97 (0.84–1.13) |  |  |
| DBP | 0.76 (0.67–0.86)* |  |  |  | 0.90 (0.77–1.05) |  |  |
| BMI | 0.84 (0.74–0.96)* |  |  |  |  |  |  |
| Waist:Hip Ratio | 1.39 (1.23–1.57)* |  |  |  |  |  |  |
| Y1 Change eGFR | 1.00 (0.86–1.15) |  |  |  |  | 0.95 (0.80–1.12) |  |
| Y1 Change SBP | 0.89 (0.77–1.04) |  |  |  |  |  |  |
| Y1 Change DBP | 0.96 (0.83–1.12) |  |  |  |  |  |  |
| p value <0.05*  eGFR calculated using MDRD equation. All variables measured at baseline unless stated  Relative hazards are expressed per 1 standard deviation increase in the independent variable | | | | | | | |
