# Supplementary material for: Chronic Kidney Disease in Primary Care: Outcomes after Five Years in a Prospective Cohort Study
Source: PLoS Med. 2016 Sep 20;13(9):e1002128. doi: 10.1371/journal.pmed.1002128 (PMC5029805; doi:10.1371/journal.pmed.1002128)
Supplement: S4 Table — (DOCX) [file pmed.1002128.s008.docx]

S4 Table : Univariable and Multivariable associations of CKD remission using the MDRD equation to calculate eGFR

| Variable | Univariable Odds Ratio  (95% CI) | Multivariable Odds Ratio  (95% CI) | | | | | |
| --- | --- | --- | --- | --- | --- | --- | --- |
|  |  | Model 1  (Basic Model) | Model 2 | Model 3 | Model 4 | Model 5 | Model 6  (Best Model) |
| eGFR | 4.36 (3.54–5.37)* | 3.98 (3.22–4.93)* | 4.01 (3.23–4.98)* | 3.62 (2.88–4.56)* | 4.04 (3.26–5.02)* | 4.91 (3.87–6.22)* | 4.76 (3.75–6.05)* |
| Age | 0.66 (0.58–0.75)* | 0.77 (0.65–0.90)* | 0.77 (0.66–0.90)* | 0.81 (0.69–0.96)* | 0.79 (0.66–0.95)* | 0.82 (0.70–0.97)* | 0.84 (0.72–1.00)* |
| Female Gender | 1.88 (1.42–2.51)* | 1.23 (0.88–1.71) | 1.22 (0.87–1.71) | 1.50 (0.99–2.25) | 1.19 (0.85–1.66) | 1.20 (0.86–1.68) | 1.44 (1.00–2.07) |
| Log uACR | 0.65 (0.57–0.74)* | 0.76 (0.65–0.89)* | 0.76 (0.65–0.89)* | 0.77 (0.65–0.91)* | 0.76 (0.65–0.90)* | 0.77 (0.66–0.91)* | 0.77 (0.66–0.91)* |
| Haemoglobin | 1.38 (1.20–1.58)* |  |  | 1.32 (1.08–1.62)* |  |  | 1.27 (1.05–1.53)* |
| Phosphate | 1.02 (0.89–1.16) |  |  | 1.08 (0.91–1.27) |  |  |  |
| Corrected Calcium | 1.09 (0.96–1.25) |  |  | 1.04 (0.88–1.23) |  |  |  |
| Bicarbonate | 1.24 (1.08–1.42)* |  |  | 1.07 (0.90–1.27) |  |  |  |
| Albumin | 1.24 (1.08–1.42)* |  |  | 1.11 (0.93–1.32) |  |  |  |
| Total Cholesterol | 1.11 (0.98–1.26) |  |  | 0.85 (0.72–1.01) |  |  |  |
| Urate | 0.51 (0.44–0.60)* |  |  | 0.87 (0.71–1.07) |  |  |  |
| Diabetes | 0.55 (0.37–0.82)* |  | 1.10 (0.69–1.75) |  |  |  |  |
| Previous CVD | 0.70 (0.49–1.01) |  | 0.88 (0.58–1.36) |  |  |  |  |
| Current or ex-smoker | 0.85 (0.65–1.10) |  | 1.02 (0.75–1.39) |  |  |  |  |
| SBP | 0.81 (0.70–0.94)* |  |  |  | 0.87 (0.70–1.07) |  |  |
| DBP | 1.16 (1.02–1.33)* |  |  |  | 0.99 (0.81–1.21) |  |  |
| BMI | 0.91 (0.79–1.04) |  |  |  |  |  |  |
| Waist:Hip Ratio | 0.72 (0.63–0.83)* |  |  |  |  |  |  |
| Y1 Change eGFR | 1.17 (1.03–1.33)* |  |  |  |  | 1.62 (1.37–1.91)* | 1.61 (1.37–1.91)* |
| Y1 Change SBP | 1.12 (0.98–1.29) |  |  |  |  |  |  |
| Y1 Change DBP | 1.10 (0.96–1.26) |  |  |  |  |  |  |
| p value <0.05*  eGFR calculating using MDRD equation. All variables measured at baseline unless stated  Odds ratios are expressed per 1 standard deviation increase in the independent variable | | | | | | | |
